# Multi-Racial Normative Data for Lobar and Subcortical Brain Volumes in Old Age: Korean and Caucasian Norms May Be Incompatible With Each Other^†^

**DOI:** 10.3389/fnagi.2021.675016

**Published:** 2021-08-03

**Authors:** Yu Yong Choi, Jang Jae Lee, Kyu Yeong Choi, Uk-Su Choi, Eun Hyun Seo, IL Han Choo, Hoowon Kim, Min-Kyung Song, Seong-Min Choi, Soo Hyun Cho, Youngshik Choe, Byeong C. Kim, Kun Ho Lee

**Affiliations:** ^1^Gwangju Alzheimer's Disease and Related Dementia Cohort Research Center, Chosun University, Gwangju, South Korea; ^2^Biomedical Technology Center, Chosun University Hospital, Gwangju, South Korea; ^3^Department of Neuropsychiatry, Chosun University School of Medicine and Hospital, Gwangju, South Korea; ^4^Department of Neurology, Chosun University School of Medicine and Hospital, Gwangju, South Korea; ^5^Department of Neurology, Chonnam National University Medical School and Hospital, Gwangju, South Korea; ^6^Korea Brain Research Institute, Daegu, South Korea; ^7^Department of Biomedical Science, Chosun University, Gwangju, South Korea; ^8^Neurozen Inc., Seoul, South Korea

**Keywords:** aging, norm, ethnic difference, Alzheimer's disease, brain magnetic resonance imaging

## Abstract

Brain aging is becoming an increasingly important topic, and the norms of brain structures are essential for diagnosing neurodegenerative diseases. However, previous studies of the aging brain have mostly focused on Caucasians, not East Asians. The aim of this paper was to examine ethnic differences in the aging process of brain structures or to determine to what extent ethnicity affects the normative values of lobar and subcortical volumes in clinically normal elderly and the diagnosis in multi-racial patients with Alzheimer's disease (AD). Lobar and subcortical volumes were measured using FreeSurfer from MRI data of 1,686 normal Koreans (age range 59–89) and 851 Caucasian, non-Hispanic subjects in the ADNI and OASIS datasets. The regression models were designed to predict brain volumes, including ethnicity, age, sex, intracranial volume (ICV), magnetic field strength (MFS), and MRI scanner manufacturers as independent variables. Ethnicity had a significant effect for all lobar (|β| > 0.20, *p* < 0.001) and subcortical regions (|β| > 0.08, *p* < 0.001) except left pallidus and bilateral ventricles. To demonstrate the validity of the z-score for AD diagnosis, 420 patients and 420 normal controls were selected evenly from the Korean and Caucasian datasets. The four validation groups divided by race and diagnosis were matched on age and sex using a propensity score matching. We analyzed whether and to what extent the ethnicity adjustment improved the diagnostic power of the logistic regression model that was built using the only z-scores of six regions: bilateral temporal cortices, hippocampi, and amygdalae. The performance of the classifier after ethnicity adjustment was significantly improved compared with the classifier before ethnicity adjustment (ΔAUC = 0.10, *D* = 7.80, *p* < 0.001; AUC comparison test using bootstrap). Korean AD dementia patients may not be classified by Caucasian norms of brain volumes because the brain regions vulnerable to AD dementia are bigger in normal Korean elderly peoples. Therefore, ethnicity is an essential factor in establishing normative data for regional volumes in brain aging and applying it to the diagnosis of neurodegenerative diseases.

## Findings

1. Brain structures of cognitively normal people mostly decayed with age from 59 to 89 years old.
2. Ethnicity had a significant effect on all lobar regions and subcortical regions except left pallidus and bilateral ventricles.
3. The z-scores for brain volumes based on the prediction model incorporating ethnicity as a predictor were effective for diagnosing multi-racial patients with AD.

## Introduction

Neurodegenerative diseases, including Alzheimesr's disease (AD) dementia and other dementias, yield specific brain changes detectable by a group comparison of anatomical magnetic resonance imaging (MRI) between patients and normal controls. To measure the brain volume alternation of an individual, the normative value or reference standard is required for estimating the degree of abnormality or the deviation from the norm according to the characteristics of the person. Very few attempts had been made (Kruggel, 2006; Walhovd et al., 2011) because a large number of brain images of normal people are needed, and there are many factors to consider in producing the normative data for brain volumes using MRI: technical and physical characteristics of MRI as well as demographic and anatomical characteristics of individuals.

Recently, a series of remarkable studies for normative data (norms) of brain regions have emerged, considering almost all feasible factors (Potvin et al., 2016, 2017). However, their work missed an essential factor of racial characteristics, so they produced practically the norms for Caucasians only. Neuroanatomical differences in brain structures between Asians and Caucasians have been reported (Zilles et al., 2001; Tang et al., 2010; Chee et al., 2011). Thus, ethnicity or race should be a factor considered for producing norms of brain regions.

Particularly, the norms specific to the elderly encompassing Asians as well as Caucasians are becoming increasingly necessary. According to the United States Census Bureau (2020), people over 65 years old are 730 million people and under 10% of the world population in 2020. By 2050, the older population is expected to reach 1.6 billion. Older Asians are now 414 million, or more than half (56.8%) of the older population, and are projected to more than double to 967 million by 2050. Most researchers in the field of aging brain did not consider ethnic backgrounds and examined Western samples with a high percentage of white people. Existing findings are largely a reflection of the White or Caucasian (Resnick et al., 2003; Scahill et al., 2003; Sowell et al., 2003; Ledig et al., 2018). Moreover, the norms specific to a narrow age range have two advantages. Even with the same number of samples, the prediction model can provide more reliable and precise estimates. The predictive model could be kept simple and non-over-fitted since the relationship between age and volume can be assumed to be linear.

Moreover, racial or regional differences have long been known in the cranial cavity or intracranial space among Asia, America, Europe, Oceania, and Africa (Beals et al., 1984; Howells, 1990; Rushton, 2000). Head shape has long been documented to be different between Caucasian and East Asian populations (Ball et al., 2010). The racial comparison demonstrated that East Asians have a rounder head with a flatter back and forehead than Caucasians. The shape of the head or cranium considerably determines the morphometry of the brain. It means that the normative values of brain structures could vary across ethnic populations and that the norms that take into account ethnicity are needed.

The study aimed to present the normative data of lobar and subcortical brain volumes for both the Asians and Caucasian elderly and determine whether and to what extent ethnicity affects the volumes in normal brain aging. To this end, we selected brain MRIs of 1,686 cognitively normal (CN) elderly people from the Gwangju Alzheimer's and Related Dementia (GARD) cohort in the Republic of Korea. For a Caucasian sample, we collected 851 brain images from the AD Neuroimaging Initiative (ADNI) and Open Access Series of Imaging Studies (OASIS) datasets. Our methods differ in detail but followed the procedures outlined in Potvin et al. (2016). We estimated lobar and subcortical volumes using FreeSurfer, an automated segmentation software widely used in neuroimaging research and created prediction models for each brain region's volume according to ethnicity, age, sex, ICV, scanner manufacturer, MFS.

The z-score as the difference between expected and actual volumes allows testing each brain structure for volume abnormality and the effect size. Finally, our objective was to determine whether ethnicity as a predictive variable resulted in substantially improved diagnosis performance when the normative z-score was applied to patients with AD; for this purpose, we analyzed additionally 420 images from patients with AD and compared the z-scores and the classifiers before and after ethnicity adjustment in the area under the receiver operating characteristics curve (AUC).

## Methods

### Normative Samples for Koreans and Caucasians

#### Koreans

The study protocol was approved by the institutional review board of Chosun University Hospital, Republic of Korea. All volunteers or the next of kin of patients gave written informed consent before participation. They were registered in the GARD cohort by GARD Cohort Research Center at Gwangju City, Republic of Korea, from April 2010 to March 2018.

A normative sample for Koreans aged 59–89 years was included from the Korean elderly cohort in this study. All participants were evaluated by comprehensive interviews, neurological examinations, and neuropsychological tests. Neuropsychological tests consist of the Korean version of mini-mental state examination (K-MMSE) (Folstein et al., 1975), Clinical Dementia Rating (CDR) (Morris, 1993), and Seoul Neuropsychological Screening Battery (SNSB) (Kang et al., 2012). The exclusion criteria for all subjects were the presence of a focal lesion on brain MRI, history of head trauma, or psychiatric disorders that could affect their mental function. Individuals with minor medical abnormalities were included.

#### Caucasians

**Figure 1:**
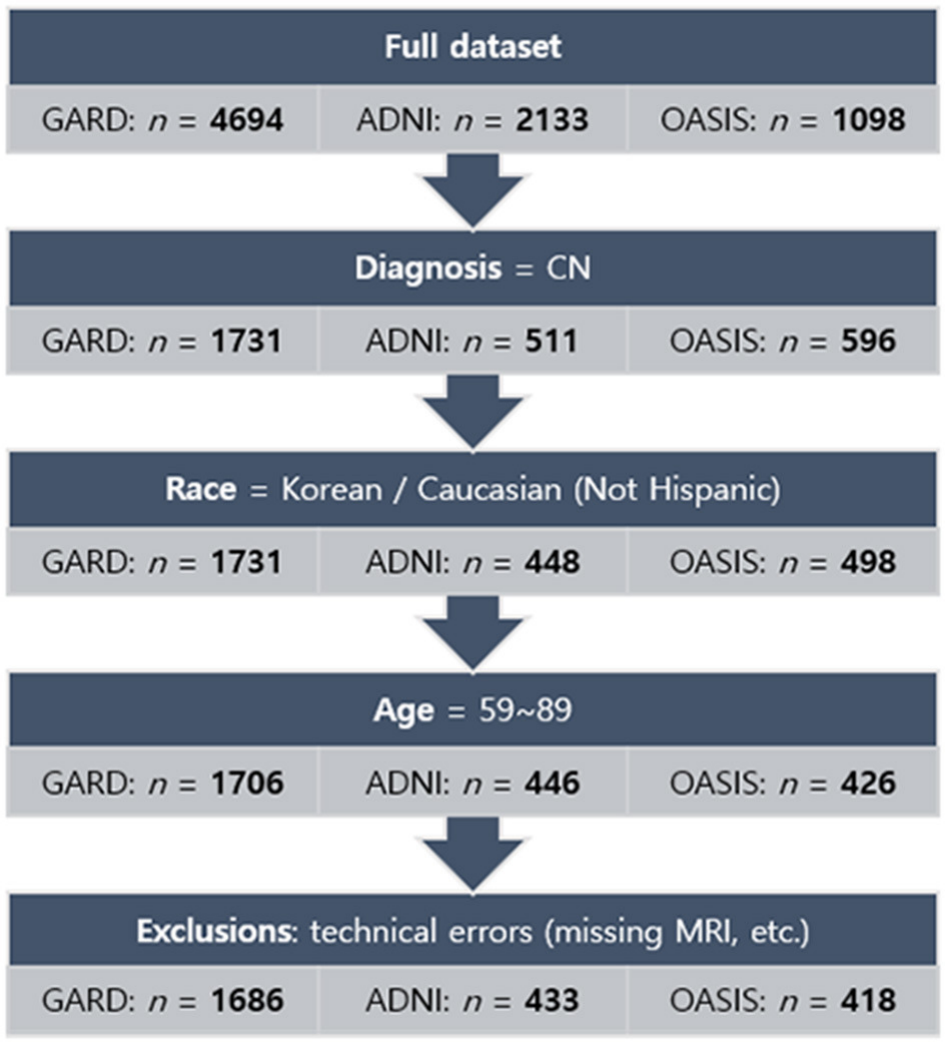
Flowchart of the process of subject selection for the norms. The normative sample finally comprised 2,537 subjects: 1,686 for Koreans and 851 for Caucasians. GARD, Gwangju Alzheimer's and Related Dementia. ADNI, Alzheimer's Disease Neuroimaging Initiative. OASIS, Open Access Series of Imaging Studies.

To investigate ethnic differences, we collected Caucasians excluding Hispanic subjects (851 CN cases) from the ADNI database (http://adni.loni.usc.edu) and the OASIS project (https://www.oasis-brains.org). The age range was matched with our dataset (59–89 years). Figure 1 showed the inclusion and exclusion criteria. In more technical detail, for ADNI, we applied one of the search conditions per step: VISCODE = “bl” (step 1) AND DX = “CN” (step 2) AND PTRACCAT = “White” AND PTETHCAT = “Not Hisp/Latino” (step 3) AND age > 59 AND age < 90 (step 4). Thirteen subjects were excluded in the final step. For OASIS, the search conditions were Visit ID = “d000” or the first date of MRI scans (step 1) AND DX1 = “Cognitively normal” (step 2) AND Race = “Caucasian” AND Ethnicity = “Non-Hispanic” (step 3) AND age > 59 AND age < 90 (step 4). Eight subjects were excluded in the final step.

Finally, the study sample for analysis comprised 2,537 subjects whose demographic information was described in Table 1.

**Table 1:** Cohort sizes and demographics for normal Koreans and Caucasians.

					**Age (y)**			**MMSE**		**Education (y)**	
**Race**	**Dataset**	***n***	**F**	**3T**	**Range**	**Mean**	**SD**	**Mean**	**SD**	**Mean**	**SD**
Korean	GARD	1686	62%	81%	59–89	73.1	5.5	27.0	2.1	9.6	4.6
Caucasian	ADNI, OASIS	851	55%	77%	59–89	73.2	6.3	29.1	1.1	16.3	2.6

*GARD, Gwangju Alzheimer's Disease and Related Dementias; ADNI, Alzheimer's Disease Neuroimaging Initiative; OASIS, Open Access Series of Imaging Studies. F, female; 3T, MRI image using a 3 Tesla scanner; y, years; MMSE, mini mental state examination*.

Some of the Caucasian data used in the preparation of this article were obtained from the ADNI database (adni.loni.usc.edu). The ADNI was launched in 2003 as a public-private partnership led by Principal Investigator Michael W. Weiner, MD. The primary goal of ADNI has been to test whether serial magnetic resonance imaging (MRI), positron emission tomography (PET), other biological markers, and clinical and neuropsychological assessment can be combined to measure the progression of mild cognitive impairment (MCI) and early Alzheimer's disease (AD). For up-to-date information, see www.adni-info.org.

### MRI Acquisition

The brain MRI images of Korean subjects were acquired using a 3.0 T scanner (Skyra, Siemens; 20-channel head coil; MPRAGE sagittal view; TR = 2,300 ms; TE = 2.143 ms; TI = 900 ms; FA = 9°; FoV = 256 mm × 256 mm; matrix = 320 × 320; slice thickness = 0.8 mm) and a 1.5 T scanner (Avanto, Siemens; 12-channel head coil; MPRAGE axial view; TR = 1,800 ms; TE = 3.43 ms; TI = 1,100 ms; FA = 15°; FoV = 224 mm × 224 mm; matrix = 256 × 256; slice thickness = 0.9 mm) at Chosun University Hospital, Gwangju, Republic of Korea.

The brain images of Caucasians were selected with slice thickness ≤ 1.2 mm from ADNI and OASIS datasets. Since there are no sub-millimeter resolution images (voxel size < 1 mm^3^) in the ADNI dataset, we selected 1- or near-millimeter resolution images (voxel size = 1−2 mm^3^, 0.93–1.30 × 0.93–1.30 × 1.0–1.2 mm) of Caucasian brains, which were scanned at multiple centers. The MRI scanner protocols were described in detail according to each scanner model at the ADNI site (https://adni.loni.usc.edu/methods/documents/mri-protocols/) and the OASIS site (https://www.oasis-brains.org/files/OASIS-3_Imaging_Data_Dictionary_v1.5.pdf).

#### Measurement of Cortical and Subcortical Volumes

The volumes of cortical and subcortical structures were measured from each brain image using the standard recon-all processing pipeline of FreeSurfer version 5.3.0, which is documented and available for download online (http://surfer.nmr.mgh.harvard.edu/). Briefly, the steps of the process include intensity normalization (Sled et al., 1998), segmentation of the gray matter (GM), white matter (WM), and cerebrospinal fluid (CSF), and surface modeling for the GM/WM and GM/CSF borders (Dale et al., 1999; Fischl et al., 2001). Once the cortical models are complete, a number of deformable procedures can be performed for in further data processing and analysis, including surface inflation (Fischl et al., 1999a), registration to a spherical atlas which utilized individual cortical folding patterns to match cortical geometry across subjects (Fischl et al., 1999b), parcellation of the cerebral cortex into units based on gyral and sulcal structure (Fischl et al., 2004; Desikan et al., 2006), and creation of a variety of surface-based data including maps of curvature and sulcal depth. This method uses both intensity and continuity information from the entire three-dimensional MR volume in segmentation and deformation procedures to produce representations of cortical thickness, calculated as the closest distance from the GM/WM boundary to the GM/CSF boundary at each vertex on the tessellated surface (Fischl and Dale, 2000).

Subjects were excluded from all analyses if there were major errors in cortical and subcortical segmentation. To acquire consistent brain measures, we used the Desikan–Killiany–Tourville (DKT) atlas (Klein and Tourville, 2012), which has the advantages of having unambiguous regional definitions and boundaries well-suited to the FreeSurfer classifier algorithm.

### Statistical Analysis

All statistical analyses, including regression models predicting cortical and subcortical volumes, were conducted in R version 3.6.3 (https://www.r-project.org/). In the previous study (Potvin et al., 2016), the regression model analyses were performed using age, sex, intracranial volume (ICV), magnetic field strength (MFS), and scanner manufacturers as predictors. Quadratic and cubic terms for ICV were tested, and the following interactions: age × sex, ICV × MFS, MFS × manufacturer. For age, unlike their model including quadratic and cubic terms, our prediction models adopted only a linear term because the age range was narrow for the subjects and the relations between age and GM volume are nearly linear from adulthood (Sowell et al., 2003; Fox and Schott, 2004; Fjell et al., 2009; Salthouse, 2011). The base model before including ethnicity as a predictor was as follows:

$$\begin{array}{r} \hat{V}=\beta_{1}\cdot age+\beta_{2}\cdot sex+\beta_{3}\cdot ICV+\beta_{4}\cdot ICV^{2}\\ +\beta_{5}\cdot ICV^{3}+\beta_{6}\cdot MFS+\beta_{7}\cdot manufacturer+\beta_{8}\cdot sex\times age\\ +\beta_{9}\cdot MFS\times manufacturer+\beta_{10}\cdot MFS\times ICV\\ +\beta_{11}\cdot ICV\times manufacturer+\alpha \end{array}$$

Our final model including ethnicity as well as the interaction terms: ethnicity × age, ethnicity × sex was as follows:

$$\begin{array}{r} \hat{V}=\beta_{1}\cdot \text{e}thnicit\text{y}+\beta_{2}\cdot age+\beta_{3}\cdot sex+\beta_{4}\cdot ICV\\ +\beta_{5}\cdot ICV^{2}+\beta_{6}\cdot ICV^{3}+\beta_{7}\cdot MFS+\beta_{8}\cdot manufacturer\\ +\beta_{9}\cdot \text{e}thnicity\times ag\text{e}+\beta_{10}\cdot ethnicity\times se\text{x}\\ +\beta_{11}\cdot sex\times age+\beta_{12}\cdot MFS\times manufacturer\\ +\beta_{13}\cdot MFS\times ICV+\beta_{14}\cdot ICV\times manufacturer+\alpha \end{array}$$

To prevent overfitting and boost generalizability, 10-fold cross-validation was performed on all the predictive models using the caret package. Ventricular volumes were log-transformed to analyze because of the skewed distribution, and the estimated coefficients for the ventricular volumes were back-transformed to represent cm^3^ or a % increase per year.

For the z-score distribution of normal controls and AD subjects of Koreans and Caucasians, the four groups were matched to each other based on age, sex, and MFS using a propensity score matching method of MatchIt package in R (see Table 4 for the details of the four groups). The normal controls matched to patients with AD were selected again from the aforementioned normative samples.

#### Normative Statistics

We turn to the calculations required to draw inferences concerning the discrepancies between a given subject's obtained volume, *V*_0_, and the volume predicted by the regression model, $\hat{V}$. The following methods are those developed by Crawford, Garthwaite (Crawford et al., 2012).

The first step is to calculate the standard error (SE) of a predicted volume for a new subject, denoted as *s*_n+1_. This SE can be expressed in this form:

$$\begin{array}{l} s_{n+1}=s_{V\cdot x}\sqrt{1+\frac{1}{n}+\frac{1}{n-1}\sum r^{ii}z_{i0}^{2}+\frac{2}{n-1}\sum r^{ij}z_{i0}z_{j0}} \end{array}$$

where *s*_*V*·*x*_ represents the root mean square error (also called residual standard deviation or SE of estimate) of the model predicting normative values, *r*^*ii*^ identifies the main diagonal elements of the inverted correlation matrix (R^−1^) for the *k* predictor variables, *r*^*ij*^ identifies off-diagonal elements, and $z_{0}={\left(z_{10},...,z_{k0}\right)}^{\prime }$ identifies the subject's values on the predictor variables in *z-score* form. We use the form $z_{i0}=\left(n-1\right)\left(x_{i0}-{\bar{x}}_{i}\right)/{\Sigma \left(x_{ij}-{\bar{x}}_{i}\right)}^{2}$. The first summation is over the *k* diagonal elements, and the second is over the *k*(*k*-1)/2 off-diagonal elements below (or above) the diagonal.

For effect size, a *z-score* (z) was computed by the formula below, using the discrepancy between a subject's actual (*V*_0_) and predicted volumes ($\hat{V}$), divided by *s*_*n*+1_ the SE of the predicted volume:

(1) $$\begin{array}{r} z=\frac{V_{0}-\hat{V}}{s_{n+1}.} \end{array}$$

#### Vertex-Wise Analysis

Vertex-wise cortical volume comparisons were rendered on the cortical surface using the regression models implemented in the SurfStat toolbox (http://www.math.mcgill.ca/keith/surfstat/) in MATLAB R2016a (The Mathworks, Natick, MA, USA). A random field theory (RFT)-based correction for multiple comparisons was applied at the cluster level with *p* = 0.05 as the significance threshold.

#### Classification of Korean Patients With AD From Caucasian Normal People

The logistic regression model analyses were built using the only z-scores of six regions: bilateral temporal cortices, hippocampi, and amygdalae. The best classification model was determined with 10-fold cross-validation using the caret package. The two receiver operating curves (ROCs) and the areas under the ROC (AUCs) were calculated using the pROC package. All the bootstrap operations for the significance of AUC comparison were performed with non-parametric stratified resampling (Carpenter and Bithell, 2000), and 10,000 bootstrap replicates to obtain a good estimate of the statistics.

## Results

### Prediction Model Including Ethnicity as a Predictor

Table 2 describes fit measures and standardized coefficients of the models predicting lobar and subcortical volumes of the Korean and Caucasian subjects (*n* = 2,537). The models for subcortical GM volumes explained considerate portions of the variance (mean *R*^2^: 31.8%, range: 12.7–45.5%). The models for lobar GM volumes explained more (mean *R*^2^: 54.0%, range: 36.1–65.5%). As shown in Table 2, ethnicity had a substantial effect for all regions except left pallidus and bilateral ventricles, and age also had a substantial effect for all regions except bilateral pallidus.

**Table 2:** Standardized coefficients of the prediction model of lobar and subcortical gray matter volumes.

	**Model**				**Coefficent**																
	**RMSE**		***R*** ^****2****^			**Ethnicity**	**Sex**	**Intracrainial volume**			**MFS**	**Manufacturer**		**Intraction**							
	***M***	***SD***	***M***	***SD***	**Age**	**Caucasian/ Korean**	**M/F**	**ICV**	**ICV^**2**^**	**ICV^**3**^**	**1.5T/3.0T**	**GE/ Siemens**	**Philips/ Siemens**	Ethnicity*Age.	Ethnicity*Sex	Sex*Age	MFS*GE	MFS *Philips	MFS*ICV	ICV*GE	ICV *Philips
**Brain**	44.42	1.88	0.78	0.02	***−0.29***	***−0.32***	***−0.08***	***0.94***	***0.03***	***−0.09***	***−0.26***	***0.08***	0.02	−0.01	***0.07***	**−0.03**	0.03	**0.04**	0.00	−0.01	**−0.03**
**Lobar GM**	22.14	1.10	0.71	0.03	***−0.23***	***−0.41***	***−0.06***	***0.83***	**0.03**	***−0.10***	***−0.45***	***0.09***	−0.02	−0.02	**0.05**	−0.02	0.03	***0.07***	0.00	**−0.03**	**−0.04**
Frontal L	4.45	0.19	0.61	0.04	***−0.18***	***−0.29***	**−0.06**	***0.83***	0.03	***−0.10***	***−0.36***	***0.09***	−0.03	**−0.03**	**0.04**	−0.02	−0.03	**0.05**	−0.01	**−0.03**	**−0.03**
Frontal R	4.51	0.19	0.62	0.03	***−0.19***	***−0.33***	**−0.05**	***0.83***	0.02	***−0.10***	***−0.37***	***0.10***	−0.02	−0.01	**0.04**	−0.03	−0.01	**0.05**	−0.01	**−0.03**	**−0.03**
Temporal L	3.31	0.13	0.63	0.03	***−0.33***	***−0.45***	**−0.05**	***0.73***	0.02	***−0.08***	***−0.38***	***0.12***	0.00	0.03	***0.08***	−0.02	0.03	***0.06***	0.01	**−0.03**	**−0.04**
Temporal R	3.22	0.15	0.63	0.03	***−0.30***	***−0.41***	**−0.05**	***0.76***	0.02	***−0.08***	***−0.39***	***0.11***	−0.01	0.01	***0.07***	−0.01	0.01	***0.06***	0.00	**−0.03**	−0.02
Parietal L	3.28	0.14	0.64	0.04	***−0.19***	***−0.44***	***−0.10***	***0.76***	**0.03**	***−0.11***	***−0.51***	**0.05**	**−0.04**	−0.03	**0.04**	−0.01	**0.07**	***0.09***	0.00	**−0.03**	**−0.04**
Parietal R	3.33	0.16	0.65	0.04	***−0.19***	***−0.44***	***−0.09***	***0.76***	**0.03**	***−0.10***	***−0.51***	**0.06**	**−0.05**	**−0.04**	**0.05**	−0.01	**0.05**	***0.08***	−0.01	**−0.03**	**−0.04**
Occipital L	1.98	0.08	0.50	0.04	***−0.25***	***−0.31***	−0.03	***0.59***	**0.03**	**−0.08**	***−0.50***	**0.07**	−0.03	−0.01	**0.06**	−0.01	**0.06**	***0.09***	0.01	**−0.03**	**−0.05**
Occipital R	2.01	0.08	0.51	0.04	***−0.24***	***−0.26***	0.00	***0.63***	0.02	***−0.09***	***−0.48***	0.04	**−0.04**	−0.02	**0.05**	−0.01	0.04	***0.09***	0.00	**−0.03**	**−0.04**
Cingulate L	1.02	0.04	0.46	0.03	***−0.17***	***−0.20***	−0.03	***0.74***	**0.04**	***−0.09***	***−0.30***	0.03	−0.02	−0.01	0.03	0.01	0.03	**0.05**	−0.03	−0.01	−0.03
Cingulate R	1.00	0.04	0.36	0.04	***−0.16***	***−0.31***	−0.03	***0.65***	0.01	***−0.11***	***−0.25***	***0.09***	**0.05**	0.00	0.02	0.01	−0.02	0.00	0.01	−0.02	**−0.04**
Insular L	0.45	0.02	0.41	0.04	**−0.05**	***−0.31***	0.00	***0.68***	0.01	**−0.07**	***−0.14***	***0.11***	0.02	−0.01	0.03	−0.01	−0.02	0.01	0.01	−0.01	−0.03
Insular R	0.47	0.02	0.44	0.04	**−0.07**	***−0.38***	**0.05**	***0.61***	0.01	−0.05	***−0.19***	**0.06**	0.01	0.01	0.03	−0.01	0.00	0.02	**0.04**	−0.01	−0.02
**Subcortical GM**	3.10	0.13	0.53	0.04	***−0.32***	***−0.25***	−0.03	***0.68***	0.01	**−0.05**	***−0.32***	0.01	0.01	0.03	**0.06**	−0.02	0.04	**0.05**	0.03	−0.01	−0.02
Thalamus L	0.80	0.04	0.38	0.04	***−0.13***	***−0.47***	**−0.08**	***0.53***	0.01	**−0.06**	***−0.32***	0.02	0.03	−0.01	**0.06**	−0.02	***0.10***	0.04	0.01	0.00	−0.01
Thalamus R	0.53	0.02	0.46	0.04	***−0.17***	***−0.44***	**−0.05**	***0.60***	0.03	−0.04	***−0.17***	***−0.12***	0.01	−0.03	0.04	−0.02	***0.15***	0.02	0.02	−0.03	−0.01
Putamen L	0.64	0.02	0.19	0.04	***−0.24***	***0.13***	0.01	***0.33***	−0.02	0.01	***−0.17***	−0.05	−0.04	**0.06**	0.01	−0.02	0.04	0.04	0.00	0.00	−0.02
Putamen R	0.54	0.02	0.20	0.05	***−0.28***	***0.08***	**0.06**	***0.30***	−0.01	0.01	***−0.14***	0.05	−0.03	***0.08***	0.01	−0.02	**−0.07**	0.02	0.03	0.01	**−0.05**
Hippocampus L	0.39	0.02	0.34	0.04	***−0.43***	***−0.28***	**−0.05**	***0.33***	0.00	−0.06	***−0.33***	**0.06**	**0.05**	0.02	0.05	0.00	0.03	0.00	**0.04**	0.00	−0.02
Hippocampus R	0.41	0.02	0.40	0.05	***−0.43***	***−0.34***	**−0.06**	***0.34***	−0.02	−0.03	***−0.40***	0.01	**0.06**	0.04	**0.07**	0.00	**0.08**	0.03	0.03	−0.02	−0.02
Caudate L	0.39	0.01	0.20	0.04	−0.04	**−0.08**	***−0.13***	***0.54***	0.00	−0.03	0.00	0.01	−0.04	0.04	0.00	0.00	−0.04	0.04	0.01	0.00	0.02
Caudate R	0.40	0.02	0.27	0.04	**−0.07**	***0.20***	**−0.07**	***0.48***	−0.01	0.00	***0.07***	0.05	**−0.06**	**0.07**	−0.01	−0.03	***−0.11***	0.02	0.04	−0.03	0.01
Amygdala L	0.19	0.01	0.40	0.04	***−0.31***	***−0.18***	**0.06**	***0.28***	−0.01	−0.03	***−0.54***	−0.04	0.02	**0.04**	0.04	−0.01	***0.10***	**0.05**	**0.04**	−0.02	−0.02
Amygdala R	0.20	0.01	0.38	0.05	***−0.28***	***−0.20***	***0.10***	***0.33***	−0.01	−0.04	***−0.50***	***0.10***	**0.06**	**0.04**	**0.06**	−0.01	0.03	0.04	0.02	−0.02	−0.03
Pallidus L	0.22	0.01	0.13	0.03	**0.07**	−0.04	0.05	***0.30***	0.02	0.03	0.00	**−0.09**	0.01	**−0.06**	−0.03	0.00	***0.11***	0.02	0.03	−0.03	−0.01
Pallidus R	0.18	0.01	0.22	0.04	0.01	***−0.22***	**−0.06**	***0.43***	−0.01	−0.02	***−0.35***	0.05	−0.02	**−0.07**	**0.06**	0.02	***0.12***	***0.10***	0.01	0.00	−0.02
Accumbens L	0.09	0.00	0.26	0.05	***−0.38***	***0.26***	−0.01	***0.14***	−0.02	−0.04	***−0.14***	***0.16***	**−0.05**	0.02	0.05	0.01	***−0.14***	0.02	**0.06**	**−0.04**	**−0.04**
Accumbens R	0.08	0.00	0.38	0.05	***−0.32***	***0.29***	**0.07**	***0.20***	−0.02	−0.04	***−0.41***	0.02	−0.02	0.01	0.02	0.00	−0.05	**0.06**	−0.02	0.00	**−0.04**
Ventral DC L	0.29	0.01	0.44	0.04	***−0.11***	***−0.22***	0.01	***0.66***	0.02	**−0.06**	***−0.28***	0.00	0.00	0.00	***0.08***	−0.03	**0.06**	***0.07***	−0.01	−0.01	−0.03
Ventral DC R	0.26	0.01	0.45	0.04	***−0.22***	***−0.14***	0.04	***0.65***	**0.05**	**−0.08**	***−0.25***	0.00	0.00	0.01	**0.06**	0.00	0.03	**0.04**	0.02	0.00	−0.02
Stem	1.61	0.07	0.42	0.04	***−0.16***	***−0.12***	**−0.06**	***0.70***	0.02	**−0.06**	***−0.06***	**−0.07**	0.03	0.01	***0.11***	−0.03	***0.11***	0.02	0.01	−0.02	−0.03
Corpuscallosum	0.36	0.01	0.20	0.04	***−0.28***	***−0.17***	***−0.15***	***0.36***	**−0.04**	−0.03	***−0.27***	−0.01	0.02	−0.02	**0.06**	0.00	0.06	0.04	0.02	0.00	−0.02
**Ventricle**	0.15	0.01	0.45	0.04	***0.49***	0.00	0.02	***0.47***	0.00	0.01	**−0.04**	0.03	0.00	**−0.05**	−0.01	**−0.04**	0.01	0.00	0.02	0.01	0.00
Lateral L	0.16	0.01	0.42	0.04	***0.48***	−0.01	0.00	***0.47***	0.00	0.01	**−0.04**	0.02	0.00	**−0.05**	−0.01	**−0.04**	0.01	0.01	0.01	0.01	0.00
Lateral R	0.17	0.01	0.42	0.05	***0.49***	**0.04**	0.00	***0.46***	−0.01	0.01	−0.03	0.03	0.00	**−0.06**	−0.01	**−0.04**	0.00	0.00	0.03	0.00	0.01
Inferior lateral L	0.23	0.01	0.39	0.04	***0.49***	**−0.04**	***0.17***	***0.21***	0.02	0.04	−0.02	0.02	−0.02	−0.02	−0.02	−0.02	**0.06**	0.04	0.03	**−0.04**	0.00
Inferior lateral R	0.26	0.01	0.36	0.04	***0.48***	0.02	***0.16***	***0.23***	0.00	0.00	0.02	**0.06**	**−0.06**	***−0.07***	−0.01	0.00	0.03	0.02	**0.05**	**−0.05**	0.00
3rd	0.12	0.00	0.44	0.05	***0.41***	***−0.18***	***0.19***	***0.39***	0.01	−0.01	***−0.06***	**0.07**	0.02	−0.01	−0.04	**−0.04**	0.04	0.00	0.02	−0.03	0.00
4th	0.12	0.00	0.14	0.04	***0.14***	***−0.12***	0.05	***0.25***	−0.01	0.07	**−0.05**	0.05	−0.01	−0.01	0.05	0.00	−0.06	−0.01	−0.02	0.03	0.02

*The values with p < 0.00125 are presented in bold and italic. The values with p < 0.05 are presented in bold. L, left; R, right; MFS, magnetic field strength; GM, gray matter; DC, diencephalon*.

**Figure 2:**
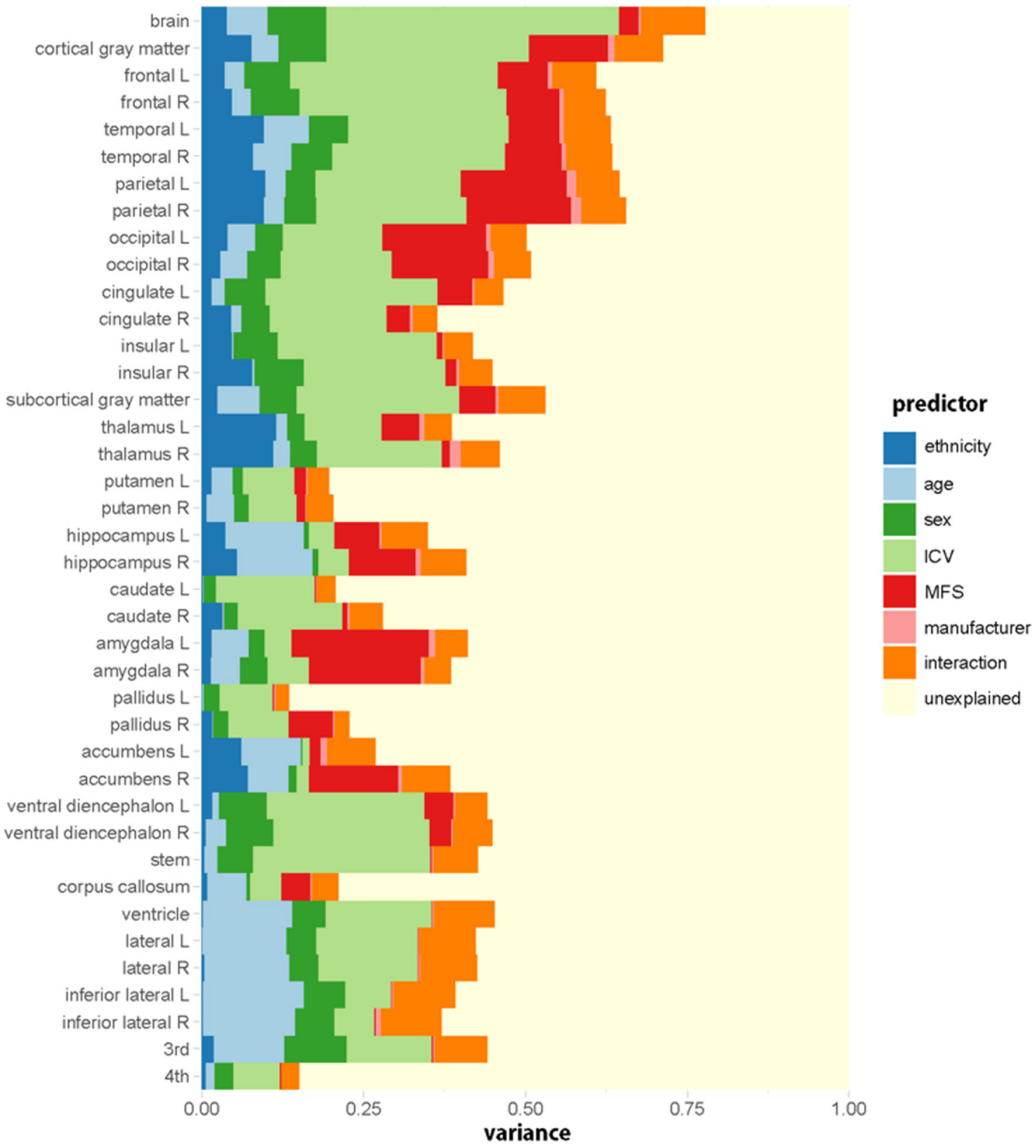
Relative importance (*R*^2^, proportion of the variance explained) of each predictive variable in the regression model for each regional volume. Ethnicity (dark blue) has a substantial effect on brain volumes. The relative importance is computed by averaging each predictor's explained proportion of the variance over all orderings of predictors. Interaction indicates the sum of the proportions of variance explained by all the interaction terms.

Figure 2 shows the relative importance or explained variance predicted by each predictor (for detailed results, see Table 3). Focusing on ethnicity and age, the two main variables of interest, lobar volumes, were largely predicted by ethnicity (mean *R*^2^: 5.9%, range: 1.6–9.9%) compared with age (mean *R*^2^: 3.1%, range: 0.3–7%) whereas subcortical volumes were largely predicted by age (mean *R*^2^: 4%, range: 0–12.2%) compared with ethnicity (mean *R*^2^: 3.5%, range: 0.1–11.6%). The effects of ethnicity on the brain volumes were comparable to those of age. Even in some modified models, ethnic effects were substantial (Supplementary Tables 2, 3).

**Table 3:** Percentage of the variance explained by each predictor in models predicting lobar and subcortical regional volumes.

	**Ethnicity**	**Age**	**Sex**	**ICV**	**MFS**	**Manufacturer**	**Interaction**	**Unexplained**
**Brain**	4.0	6.2	9.1	45.3	3.0	0.3	10.0	22.1
**Cortical gray matter**	7.7	4.2	7.4	31.3	12.3	1.0	7.5	28.7
Frontal L	3.6	3.0	7.2	32.0	7.8	0.7	6.8	39.0
Frontal R	4.7	2.9	7.5	32.0	8.2	0.7	6.5	37.5
Temporal L	9.6	7.0	6.0	24.9	7.8	0.7	7.3	36.7
Temporal R	8.0	6.0	6.3	26.7	8.7	0.7	7.1	36.5
Parietal L	9.9	3.1	4.7	22.4	16.3	1.5	6.8	35.3
Parietal R	9.6	3.1	5.0	23.2	16.2	1.5	7.0	34.4
Occipital L	4.0	4.3	4.2	15.4	16.1	0.7	5.5	49.8
Occipital R	2.9	4.1	5.2	17.2	15.0	0.8	5.7	49.1
Cingulate L	1.6	1.9	6.3	26.6	5.3	0.3	4.5	53.3
Cingulate R	4.6	1.6	4.3	18.0	3.6	0.4	3.8	63.5
Insular L	4.7	0.3	6.8	24.6	0.8	0.3	4.4	58.1
Insular R	7.8	0.4	7.7	21.8	1.7	0.5	5.1	55.1
**Subcortical gray matter**	2.5	6.5	5.7	25.2	5.6	0.5	7.3	46.8
Thalamus L	11.6	1.6	2.7	11.9	5.9	0.7	4.3	61.2
Thalamus R	11.1	2.5	4.2	19.3	1.3	1.7	6.0	53.9
Putamen L	1.6	3.3	1.6	8.0	1.8	0.2	3.4	80.2
Putamen R	0.8	4.3	2.2	7.4	1.4	0.1	4.2	79.6
Hippocampus L	3.7	12.2	0.7	4.0	6.9	0.4	7.1	65.0
Hippocampus R	5.5	11.7	0.9	4.7	10.4	0.8	7.0	59.1
Caudate L	0.3	0.1	1.9	15.3	0.2	0.2	2.9	79.2
Caudate R	3.2	0.3	2.1	16.1	0.8	0.3	5.2	72.0
Amygdala L	1.6	5.7	2.5	4.2	21.2	1.0	5.0	58.8
Amygdala R	1.5	4.4	4.4	6.3	17.3	0.5	4.2	61.4
Pallidus L	0.1	0.2	2.5	8.2	0.1	0.2	2.1	86.5
Pallidus R	1.7	0.0	2.4	9.3	6.9	0.2	2.3	77.2
Accumbens L	6.2	9.1	0.3	1.1	1.6	1.1	7.5	73.1
Accumbens R	7.2	6.3	1.2	2.0	13.8	0.5	7.6	61.5
Ventral diencephalon L	1.7	1.0	7.4	24.3	4.5	0.3	5.1	55.8
Ventral diencephalon R	0.7	3.1	7.3	24.0	3.4	0.2	6.2	55.0
Stem	0.4	2.1	5.4	27.4	0.2	0.3	6.9	57.3
Corpus callosum	1.0	6.0	0.5	4.9	4.5	0.3	4.2	78.7
**Ventricle**	0.2	13.8	5.2	16.4	0.1	0.1	9.5	54.7
Lateral L	0.1	13.1	4.5	15.7	0.1	0.1	8.8	57.6
Lateral R	0.5	13.1	4.4	15.4	0.1	0.2	8.8	57.4
Inferior lateral L	0.2	15.7	6.4	7.1	0.1	0.2	9.6	60.7
Inferior lateral R	0.2	14.3	6.0	6.2	0.3	0.8	9.4	62.9
3rd	1.9	10.9	9.6	13.2	0.2	0.2	8.2	55.8
4th	0.7	1.3	3.0	7.2	0.2	0.1	2.7	84.9

*L, left; R, right; ICV, intracranial volume; MFS, magnetic field strength*.

**Figure 3:**
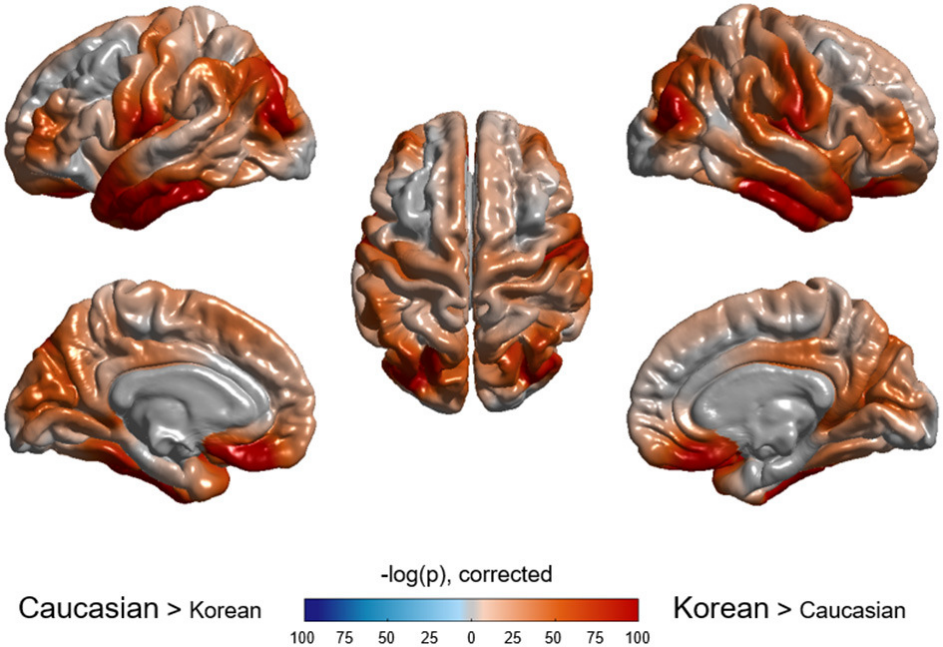
Vertex-wise comparison of cortical volume between Caucasian and Korean normal controls. Korean elderly people were bigger in cortical volume than Caucasians. The *p*-value of ethnicity as a predictor was computed at each vertex based on the regression model described in the method section.

As shown in Table 2, ethnicity had a substantial effect for all regions except left pallidus and bilateral ventricles, and age also had a substantial effect for all regions except bilateral pallidus. Cortical volumes of Koreans were larger than those of Caucasians at both lobar (Table 2) and vertex-wise levels (Figure 3). Subcortical volumes also were generally larger in Koreans, but the volumes of the putamen, accumbens, and right caudate were larger in Caucasians (Table 2).

### Lobar and Subcortical Volume Changes in Normal Aging

**Figure 4:**
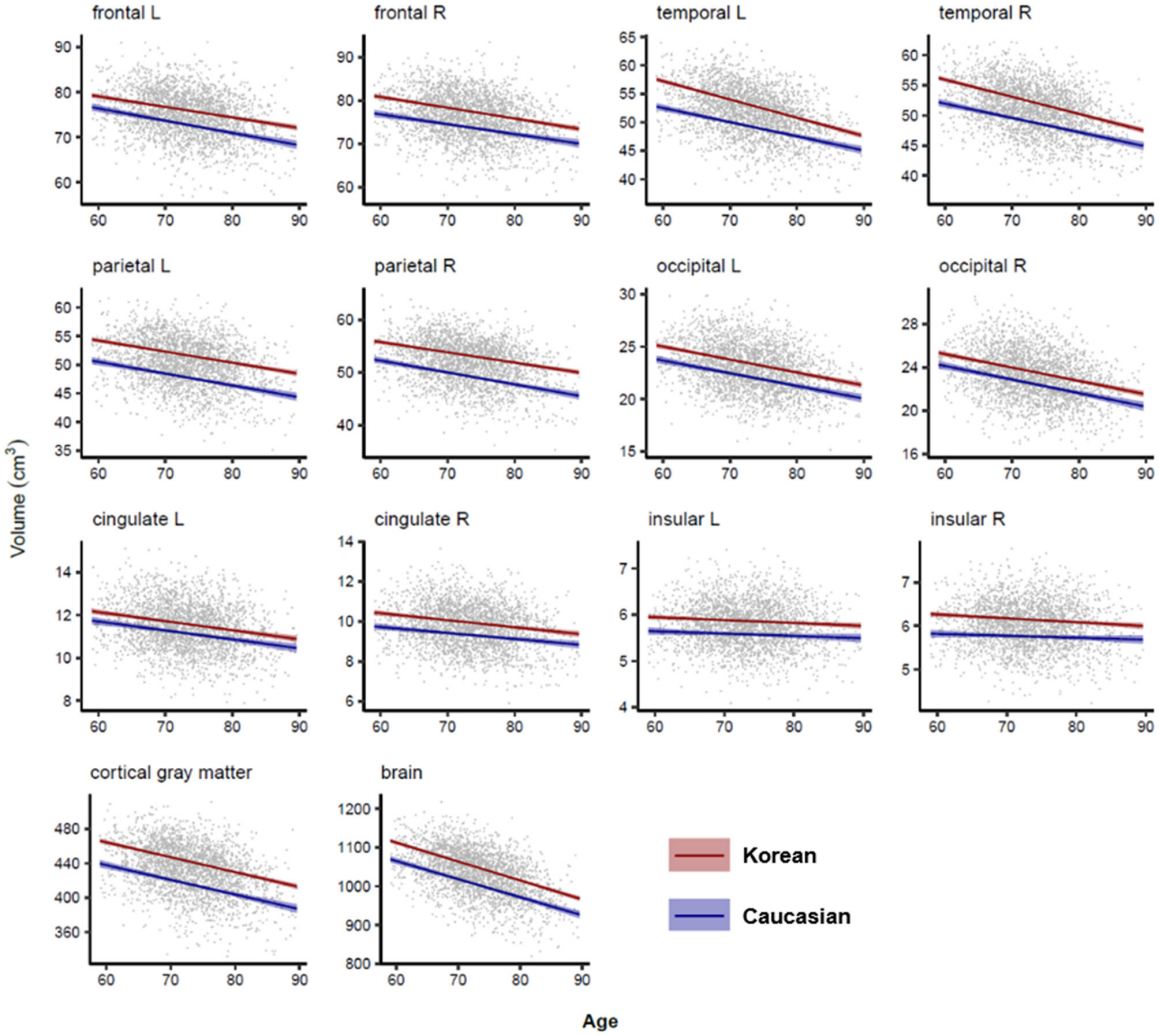
Lobar volume changes with age in Caucasian and Korean elderly people. This figure illustrates ethnic contrast on age effect in each model predicting lobar volumes in a massive sample of cognitively normal people aged 59–89 years. Each line denotes mean volume with 95% confidence intervals in the colored shade.

**Figure 5:**
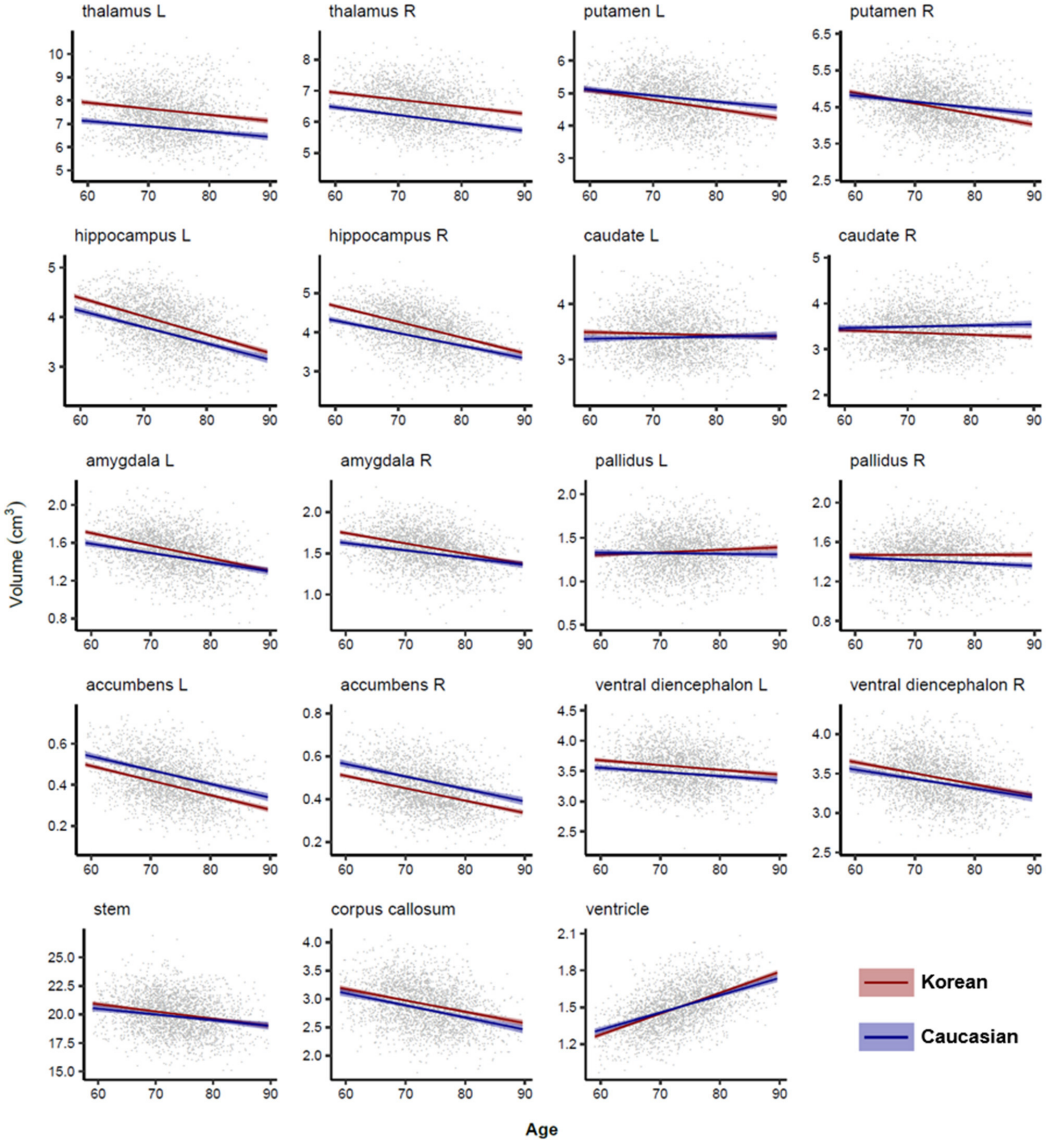
Subcortical volume changes with age in Caucasian and Korean elderly peoples. This figure illustrates ethnic contrast in age effect in each model predicting subcortical volumes in a massive sample of cognitively normal people aged 59–89 years. Each line denotes mean volume with 95% confidence intervals in the colored shade. Ventricular volumes are log_10_ transformed.

Figures 4, 5 illustrated aging slopes of lobar and subcortical regions in Caucasians and Koreans aged 59 and 89 years, or the predicted volumes for lobar and subcortical regions according to age and ethnicity. As shown in Table 2, a marked age by race interaction was found in the right putamen and right inferior lateral ventricle. Additionally, a weak age by race interaction was found in the left frontal and right parietal lobes and the left putamen, right caudate, amygdalae, pallidi, and lateral ventricles.

### Z-Scores of the Normal Controls Before/After Ethnicity Adjustment

For the validation of the z-scores based on our prediction model incorporating ethnicity, we selected Caucasian and Korean patients with AD and the matched normal controls. The four groups (two races × two diagnoses) were matched based on age, sex, and MFS (Table 4). To confirm whether the z-scores of the normal controls were close to zero, we calculated the z-scores of normal controls for Koreans and Caucasians, and the significances of the z-scores, or the differences between the observed and the predicted volumes. As shown in Table 5, the z-scores of the normal controls or the differences between the observed and the predicted volumes after ethnicity adjustment became not significant for both races. However, the differences before ethnicity adjustment were significant in most regions for either race. After ethnicity adjustment, the z-scores for lobar volumes became close to zero (mean z: −0.08, range: −0.16 to 0 for Korean; mean z: 0.03, range: −0.06 to 0.10 for Caucasian), and subcortical volumes also were close to zero (mean z: 0.02, range: −0.03 to 0.07 for Korean; mean z: 0.01, −0.11 to 0.07 for Caucasian).

**Table 4:** Sample sizes of normal people and AD patients of Koreans and Caucasians.

	**Korean**		**Korean**		**Caucasian**		**Caucasian**	
	**CN**		**AD**		**CN**		**AD**	
	**Mean**	**SD**	**Mean**	**SD**	**Mean**	**SD**	**Mean**	**SD**
*n*	210		210		210		210	
Age	75.1	5.5	74.6	6.1	75.6	5.8	74.8	6.5
Sex (M)	51.0%		50.0%		54.8%		50.0%	
Field strength (1.5T)	33.8%		37.1%		31.9%		37.6%	

*M, male; CN, normal control; AD, Alzheimer's disease*.

**Table 5:** Z-scores and the differences between the observed volumes and the predicted volumes.

	**Before ethnicity adjustment**						**After ethnicity adjustment**					
	**Kor.CN**			**Cau.CN**			**Kor.CN**			**Cau.CN**		
	***z***	***t***	***p***	***z***	***t***	***p***	***z***	***t***	***p***	***z***	***t***	***p***
**Brain**	0.24	1.59	0.113	**−0.58**	−3.11	0.002	−0.02	−0.12	0.901	−0.02	0.35	0.728
**Cortical gray matter**	0.18	1.37	0.171	***−0.62***	−4.15	0.000	−0.14	−1.03	0.304	0.04	0.59	0.555
Frontal L	0.08	0.67	0.502	**−0.37**	−2.83	0.005	−0.13	−1.10	0.270	0.08	0.56	0.574
Frontal R	0.12	0.99	0.321	***−0.39***	−3.37	0.001	−0.11	−0.91	0.365	0.10	0.37	0.710
Temporal L	0.20	1.75	0.080	***−0.61***	−3.93	0.000	−0.08	−0.70	0.482	−0.01	1.27	0.204
Temporal R	0.19	1.57	0.117	***−0.56***	−3.76	0.000	−0.08	−0.75	0.453	0.01	1.04	0.297
Parietal L	0.17	1.45	0.149	***−0.71***	−5.43	0.000	−0.16	−1.30	0.196	−0.06	0.29	0.771
Parietal R	0.18	1.59	0.112	***−0.69***	−5.17	0.000	−0.15	−1.11	0.268	−0.04	0.32	0.745
Occipital L	0.12	0.97	0.333	**−0.34**	−3.20	0.001	−0.06	−0.81	0.419	0.06	0.54	0.588
Occipital R	0.11	1.05	0.294	**−0.34**	−2.62	0.009	−0.05	−0.47	0.636	0.00	0.44	0.662
Cingulate L	0.11	1.09	0.278	**−0.30**	−2.48	0.014	−0.01	−0.12	0.901	−0.03	−0.13	0.898
Cingulate R	0.07	0.70	0.482	***−0.33***	−3.99	0.000	−0.11	−1.25	0.212	0.06	0.14	0.885
Insular L	**0.18**	2.01	0.046	***−0.31***	−3.50	0.001	0.00	0.05	0.959	0.09	0.29	0.775
Insular R	**0.21**	2.44	0.015	***−0.40***	−4.88	0.000	0.00	0.07	0.945	0.08	−0.09	0.932
**Subcortical gray matter**	0.16	1.53	0.127	**−0.33**	−3.19	0.002	0.03	0.31	0.754	−0.03	−0.42	0.672
Thalamus L	**0.25**	2.90	0.004	***−0.58***	−8.17	0.000	0.00	−0.31	0.756	−0.04	−0.95	0.344
Thalamus R	**0.28**	2.97	0.003	***−0.56***	−7.53	0.000	0.02	−0.14	0.890	0.00	−1.02	0.307
Putamen L	−0.08	−0.53	0.598	**0.13**	2.45	0.015	0.01	0.58	0.563	−0.05	−0.13	0.897
Putamen R	0.00	0.19	0.848	0.20	1.64	0.103	0.07	1.06	0.288	0.06	−0.12	0.901
Hippocampus L	**0.19**	2.21	0.028	**−0.25**	−2.73	0.007	0.06	0.58	0.564	0.06	0.74	0.457
Hippocampus R	**0.21**	2.16	0.031	***−0.34***	−3.81	0.000	0.05	0.40	0.689	0.04	0.45	0.650
Caudate L	0.01	0.43	0.670	−0.19	−1.35	0.178	−0.03	0.04	0.970	−0.11	−0.34	0.737
Caudate R	−0.08	−0.65	0.517	**0.20**	2.96	0.003	0.05	0.76	0.450	−0.07	−0.12	0.902
Amygdala L	0.13	1.05	0.293	−0.20	−1.23	0.219	0.04	0.20	0.843	0.00	0.92	0.360
Amygdala R	0.08	0.45	0.657	−0.17	−1.45	0.148	0.00	−0.48	0.628	0.03	0.77	0.442
Pallidus L	0.03	0.55	0.580	**−0.06**	−2.20	0.029	−0.02	−0.10	0.923	0.02	−1.05	0.297
Pallidus R	0.15	1.94	0.053	**−0.22**	−3.21	0.001	0.04	0.48	0.628	0.02	0.29	0.769
Accumbens L	−0.15	−1.61	0.108	***0.40***	5.73	0.000	0.03	0.81	0.419	0.02	1.29	0.199
Accumbens R	**−0.21**	−2.00	0.047	***0.48***	4.45	0.000	−0.02	0.28	0.777	0.07	−0.05	0.964
Ventral diencephalon L	0.09	1.13	0.261	−0.23	−1.88	0.061	−0.01	0.09	0.930	0.00	0.32	0.751
Ventral diencephalon R	0.08	0.93	0.355	−0.10	−1.47	0.143	0.02	0.33	0.739	0.05	−0.11	0.912
Stem	0.08	0.95	0.344	−0.03	0.18	0.859	0.06	0.76	0.450	0.03	0.76	0.450
Corpus callosum	0.11	1.35	0.179	−0.06	−1.02	0.307	0.04	0.45	0.656	0.10	0.97	0.332
**Ventricle**	−0.05	−0.78	0.438	0.00	0.34	0.731	−0.07	−0.96	0.335	0.02	0.56	0.577
Lateral L	−0.03	−0.61	0.542	0.00	0.00	0.997	−0.06	−0.91	0.365	0.05	0.42	0.673
Lateral R	−0.07	−0.94	0.346	0.04	1.11	0.266	−0.07	−0.90	0.366	0.02	0.87	0.387
Inferior lateral L	−0.07	−0.82	0.414	−0.11	−0.85	0.398	−0.11	−1.26	0.210	−0.02	0.10	0.922
Inferior lateral R	−0.08	−0.99	0.325	−0.08	−0.43	0.665	−0.09	−1.07	0.284	−0.07	−0.45	0.654
3rd	0.08	0.71	0.480	***−0.33***	−3.44	0.001	−0.06	−0.60	0.546	−0.03	−0.45	0.652
4th	−0.06	−0.73	0.469	−0.20	−1.73	0.084	−0.10	−1.35	0.177	−0.09	−0.47	0.636

*The z scores with p < 0.00125 are presented in bold and italic. The scores with p < 0.05 are presented in bold. L, left; R, right. Kor, Korean; Cau, Caucasian; CN, normal controls*.

### Crossover Classification of Korean Patients With AD From Caucasian Normal People

**Figure 6:**
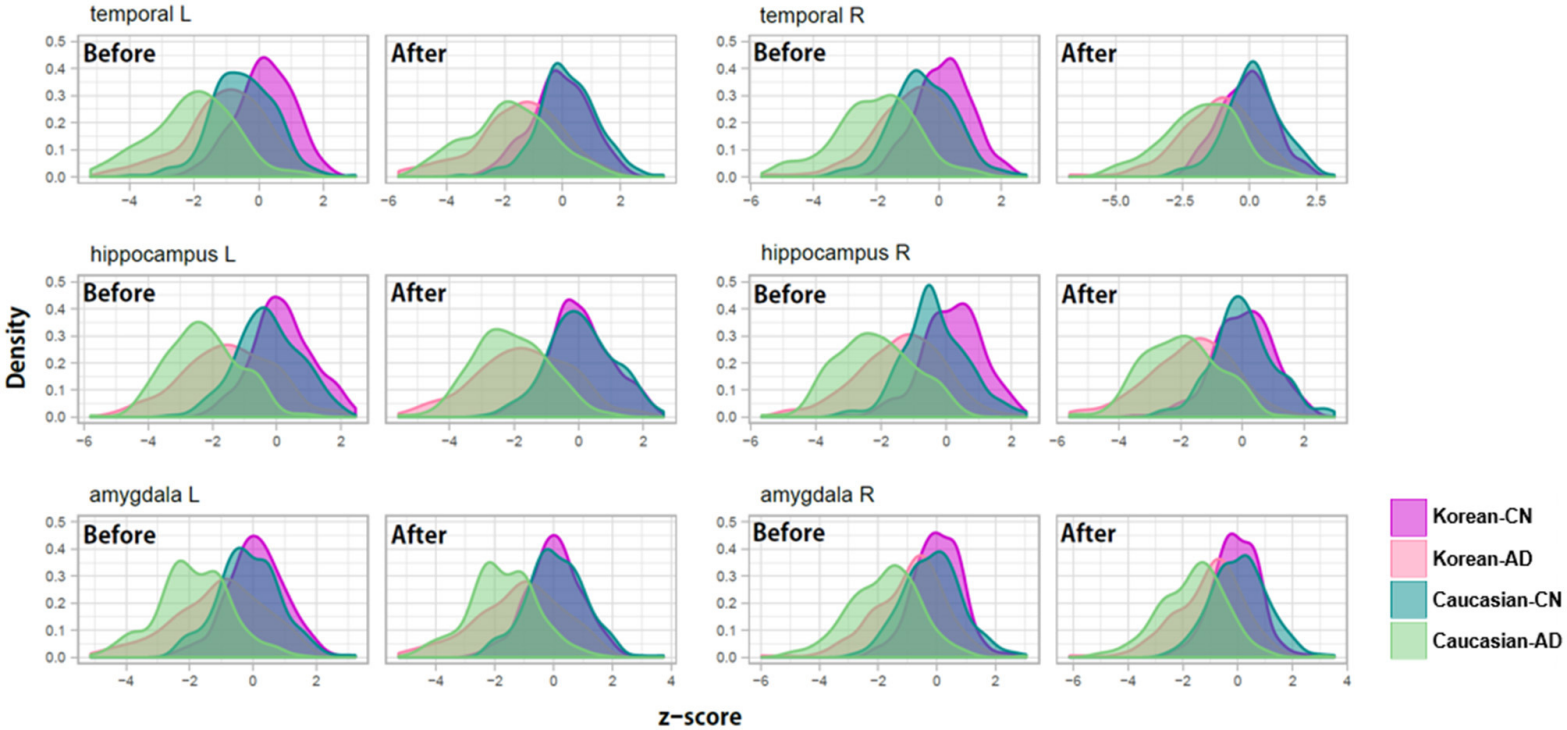
Examples of z-score distributions of patients with Alzheimer's disease and normal controls before/after adjustment for ethnic differences. Before the adjustment, the z-score distributions of each diagnosis group were separated between Koreans and Caucasians and then overlapped after the adjustment.

To verify the usefulness of our z-scoring system at diagnosis, we compared the z-scores of patients with AD and the normal controls (Table 6) and depicted the distributions of the three (bilaterally six) representative regions in Figure 6. The representative regions, the temporal cortices, hippocampi, and amygdalae were selected based on the criteria that all the four types of Δz values of each region were lesser than −1 after ethnicity adjustment (Table 6). The regions such as ventricles were not selected because they were not much affected by ethnicity.

**Table 6:** Z-score differences between AD patients and controls before/after ethnicity adjustment.

	**Before ethnicity adjustment**												**After ethnicity adjustment**											
	**Kor.AD vs. Kor.CN**			**Cau.AD vs. Kor.CN**			**Kor.AD vs. Cau.CN**			**Cau.AD vs. Cau.CN**			**Kor.AD vs. Kor.CN**			**Cau.AD vs. Kor.CN**			**Kor.AD vs. Cau.CN**			**Cau.AD vs. Cau.CN**		
	***Δz***	***t***	***p***	***Δz***	***t***	***p***	***Δz***	***t***	***p***	***Δz***	***t***	***p***	***Δz***	***t***	***p***	***Δz***	***t***	***p***	***Δz***	***t***	***p***	***Δz***	***t***	***p***
**Brain**	***−0.92***	−10.43	0.000	***−1.75***	−18.53	0.000	−0.16	−1.59	0.113	***−0.99***	−9.42	0.000	***−1.03***	−10.26	0.000	***−1.20***	−11.49	0.000	***−1.12***	−10.35	0.000	***−1.28***	−11.52	0.000
**Cortical GM**	***−0.88***	−9.26	0.000	***−1.92***	−19.47	0.000	−0.12	−1.19	0.234	***−1.17***	−10.79	0.000	***−1.02***	−9.21	0.000	***−1.33***	−11.75	0.000	***−1.27***	−10.87	0.000	***−1.58***	−13.26	0.000
Frontal L	***−0.62***	−6.03	0.000	***−1.17***	−11.68	0.000	−0.17	−1.51	0.132	***−0.71***	−6.66	0.000	***−0.65***	−5.95	0.000	***−0.67***	−6.30	0.000	***−0.87***	−7.66	0.000	***−0.88***	−8.04	0.000
Frontal R	***−0.60***	−6.07	0.000	***−1.21***	−11.98	0.000	−0.03	−0.30	0.762	***−0.64***	−5.91	0.000	***−0.64***	−6.00	0.000	***−0.67***	−6.18	0.000	***−0.81***	−7.22	0.000	***−0.84***	−7.39	0.000
Temporal L	***−1.22***	−11.41	0.000	***−2.24***	−20.75	0.000	***−0.56***	−5.06	0.000	***−1.58***	−14.11	0.000	***−1.42***	−11.37	0.000	***−1.74***	-14.29	0.000	***−1.69***	−13.45	0.000	***−2.02***	−16.39	0.000
Temporal R	***−1.02***	−9.76	0.000	***−2.20***	−20.25	0.000	***−0.38***	−3.45	0.001	***−1.57***	−13.68	0.000	***−1.15***	−9.70	0.000	***−1.71***	−14.45	0.000	***−1.40***	−11.53	0.000	***−1.96***	−16.19	0.000
Parietal L	***−0.64***	−6.95	0.000	***−1.75***	−17.06	0.000	0.16	1.65	0.100	***−0.95***	−8.64	0.000	***−0.73***	−6.83	0.000	***−1.12***	−9.29	0.000	***−0.95***	−8.57	0.000	***−1.33***	−10.81	0.000
Parietal R	***−0.61***	−6.91	0.000	***−1.78***	−16.91	0.000	**0.20**	2.10	0.036	***−0.97***	−8.59	0.000	***−0.71***	−6.84	0.000	***−1.15***	−9.37	0.000	***−0.90***	−8.39	0.000	***−1.35***	−10.66	0.000
Occipital L	***−0.39***	−4.36	0.000	***−0.99***	−10.32	0.000	0.06	0.57	0.569	***−0.54***	−5.18	0.000	***−0.41***	−4.25	0.000	***−0.53***	−5.28	0.000	***−0.56***	−5.38	0.000	***−0.68***	−6.30	0.000
Occipital R	***−0.46***	−5.07	0.000	***−1.02***	−10.78	0.000	−0.05	−0.47	0.641	***−0.60***	−5.73	0.000	***−0.47***	−4.99	0.000	***−0.62***	−6.24	0.000	***−0.57***	−5.46	0.000	***−0.72***	−6.60	0.000
Cingulate L	***−0.58***	−5.97	0.000	***−1.01***	−10.45	0.000	−0.18	−1.86	0.064	***−0.61***	−6.29	0.000	***−0.59***	−5.90	0.000	***−0.70***	−7.09	0.000	***−0.59***	−5.94	0.000	***−0.69***	−7.14	0.000
Cingulate R	***−0.33***	−3.44	0.001	***−0.87***	−8.64	0.000	0.12	1.20	0.230	***−0.42***	−4.13	0.000	***−0.34***	−3.36	0.001	***−0.42***	−4.07	0.000	***−0.47***	−4.65	0.000	***−0.55***	−5.32	0.000
Insular L	***−0.65***	−6.71	0.000	***−1.10***	−10.75	0.000	−0.08	−0.82	0.415	***−0.54***	−4.90	0.000	***−0.67***	−6.62	0.000	***−0.65***	−6.11	0.000	***−0.70***	−6.55	0.000	***−0.68***	−6.08	0.000
Insular R	***−0.61***	−6.50	0.000	***−1.16***	−11.56	0.000	0.12	1.19	0.235	***−0.43***	−4.01	0.000	***−0.65***	−6.41	0.000	***−0.62***	−5.93	0.000	***−0.63***	−5.93	0.000	***−0.60***	−5.49	0.000
**Subcortical GM**	***−1.03***	−10.46	0.000	***−1.78***	−19.00	0.000	***−0.51***	−5.08	0.000	***−1.25***	−13.19	0.000	***−1.07***	−10.37	0.000	***−1.46***	−15.14	0.000	***−0.98***	−9.42	0.000	***−1.37***	-14.05	0.000
Thalamus L	***−0.37***	−4.47	0.000	***−0.88***	−10.44	0.000	***0.48***	5.71	0.000	−0.03	−0.39	0.699	***−0.40***	−4.33	0.000	**−0.25**	−2.71	0.007	***−0.34***	−3.74	0.000	**−0.19**	−2.11	0.035
Thalamus R	***−0.41***	−4.68	0.000	***−1.06***	−12.12	0.000	***0.50***	5.71	0.000	−0.15	−1.68	0.095	***−0.44***	−4.54	0.000	***−0.42***	−4.52	0.000	***−0.36***	−3.71	0.000	***−0.33***	−3.65	0.000
Putamen L	***−0.38***	−3.63	0.000	***−0.38***	−4.16	0.000	***−0.60***	−6.11	0.000	***−0.60***	−7.21	0.000	***−0.39***	−3.70	0.000	***−0.60***	−6.65	0.000	***−0.34***	−3.41	0.001	***−0.55***	−6.69	0.000
Putamen R	***−0.52***	−5.02	0.000	***−0.72***	−7.20	0.000	***−0.63***	−6.19	0.000	***−0.83***	−8.44	0.000	***−0.53***	−5.08	0.000	***−0.89***	−8.85	0.000	***−0.43***	−4.22	0.000	***−0.79***	−8.01	0.000
Hippocampus L	***−1.69***	−14.47	0.000	***−2.46***	−24.53	0.000	***−1.24***	−10.45	0.000	***−2.01***	−19.54	0.000	***−1.75***	−14.30	0.000	***−2.15***	−20.78	0.000	***−1.76***	−14.22	0.000	***−2.17***	−20.50	0.000
Hippocampus R	***−1.59***	−14.40	0.000	***−2.37***	−23.00	0.000	***−1.05***	−9.30	0.000	***−1.82***	−17.31	0.000	***−1.68***	−14.27	0.000	***−2.02***	−18.83	0.000	***−1.69***	−14.06	0.000	***−2.02***	−18.49	0.000
Caudate L	0.02	0.18	0.856	***−0.40***	−3.92	0.000	0.18	1.53	0.128	**−0.24**	−2.50	0.013	0.02	0.17	0.863	**−0.29**	−2.91	0.004	0.05	0.46	0.648	**−0.26**	−2.69	0.007
Caudate R	−0.13	−1.22	0.223	−0.13	−1.28	0.202	***−0.45***	−4.17	0.000	***−0.44***	−4.46	0.000	−0.15	−1.31	0.190	***−0.46***	−4.49	0.000	−0.07	−0.59	0.552	***−0.38***	−3.76	0.000
Amygdala L	***−1.16***	−9.90	0.000	***−1.97***	−19.47	0.000	***−0.93***	−7.75	0.000	***−1.74***	−16.65	0.000	***−1.18***	−9.81	0.000	***−1.75***	−16.65	0.000	***−1.25***	−10.14	0.000	***−1.82***	−17.24	0.000
Amygdala R	***−0.97***	−9.57	0.000	***−1.80***	−17.80	0.000	***−0.79***	−7.27	0.000	***−1.62***	−14.91	0.000	***−0.99***	−9.48	0.000	***−1.57***	−15.40	0.000	***−1.11***	−9.91	0.000	***−1.69***	−15.40	0.000
Pallidus L	0.14	1.52	0.129	−0.06	−0.61	0.540	***0.34***	3.67	0.000	0.15	1.60	0.111	0.15	1.57	0.116	0.04	0.49	0.626	**0.22**	2.32	0.021	0.11	1.26	0.208
Pallidus R	−0.13	−1.31	0.191	***−0.47***	−5.31	0.000	**0.25**	2.70	0.007	−0.10	−1.12	0.263	−0.12	−1.20	0.229	**−0.18**	−1.99	0.048	−0.10	−1.06	0.289	−0.16	−1.87	0.062
Accumbens L	***−0.77***	−8.31	0.000	***−0.45***	−4.50	0.000	***−1.38***	−13.47	0.000	***−1.05***	−9.74	0.000	***−0.81***	−8.47	0.000	***−0.93***	−9.06	0.000	***−0.87***	−8.26	0.000	***−0.98***	−8.85	0.000
Accumbens R	***−0.57***	−6.82	0.000	***−0.44***	−4.60	0.000	***−1.16***	−12.15	0.000	***−1.03***	−9.67	0.000	***−0.61***	−6.98	0.000	***−0.99***	−9.78	0.000	***−0.58***	−5.87	0.000	***−0.96***	−8.63	0.000
Ventral DC L	**−0.26**	−2.48	0.013	***−0.59***	−6.02	0.000	0.06	0.51	0.611	**−0.28**	−2.70	0.007	**−0.26**	−2.44	0.015	**−0.31**	−3.09	0.002	**−0.28**	−2.58	0.010	**−0.33**	−3.20	0.001
Ventral DC R	***−0.46***	−4.36	0.000	***−0.65***	−6.88	0.000	−0.20	−1.76	0.078	***−0.39***	−3.75	0.000	***−0.46***	−4.35	0.000	***−0.47***	−4.97	0.000	***−0.41***	−3.59	0.000	***−0.42***	−4.02	0.000
Stem	***−0.47***	−4.72	0.000	***−0.48***	−4.86	0.000	***−0.40***	−3.65	0.000	***−0.41***	−3.76	0.000	***−0.47***	−4.72	0.000	***−0.40***	−3.97	0.000	***−0.49***	−4.42	0.000	***−0.41***	−3.73	0.000
Corpus callosum	***−0.77***	−8.24	0.000	***−0.67***	−6.90	0.000	***−0.58***	−5.79	0.000	***−0.47***	−4.62	0.000	***−0.77***	−8.20	0.000	***−0.47***	−4.84	0.000	***−0.82***	−8.22	0.000	***−0.52***	−5.04	0.000
**Ventricle**	***1.02***	11.07	0.000	***0.97***	10.14	0.000	***0.90***	9.24	0.000	***0.86***	8.45	0.000	***1.02***	11.02	0.000	***1.00***	10.54	0.000	***0.86***	8.82	0.000	***0.84***	8.43	0.000
Lateral L	***0.93***	10.35	0.000	***0.87***	9.15	0.000	***0.87***	8.91	0.000	***0.81***	7.90	0.000	***0.93***	10.32	0.000	***0.92***	9.83	0.000	***0.80***	8.17	0.000	***0.79***	7.80	0.000
Lateral R	***0.90***	9.84	0.000	***0.91***	9.55	0.000	***0.69***	7.18	0.000	***0.70***	7.04	0.000	***0.90***	9.80	0.000	***0.88***	9.31	0.000	***0.72***	7.54	0.000	***0.70***	7.13	0.000
Inferior lateral L	***1.35***	12.97	0.000	***1.42***	14.78	0.000	***1.35***	13.33	0.000	***1.42***	15.27	0.000	***1.35***	12.99	0.000	***1.53***	16.00	0.000	***1.22***	12.00	0.000	***1.39***	15.06	0.000
Inferior lateral R	***1.30***	12.66	0.000	***1.43***	14.48	0.000	***1.25***	12.58	0.000	***1.38***	14.49	0.000	***1.31***	12.61	0.000	***1.43***	14.59	0.000	***1.24***	12.49	0.000	***1.37***	14.59	0.000
3rd	***0.75***	7.53	0.000	**0.28**	2.77	0.006	***1.17***	11.99	0.000	***0.70***	7.13	0.000	***0.78***	7.62	0.000	***0.65***	6.38	0.000	***0.76***	7.63	0.000	***0.63***	6.35	0.000
4th	***0.32***	3.34	0.001	−0.17	−1.64	0.101	***0.42***	4.11	0.000	−0.07	−0.66	0.513	***0.32***	3.38	0.001	−0.04	−0.35	0.724	**0.26**	2.57	0.010	−0.10	−0.86	0.389

*L, right; R, right; ICV, intracranial volume; MFS, magnetic field strength; Kor, Korean; Cau, Caucasian; AD, AD patients; CN, normal controls; GM, gray matter; DC, diencephalon*.

As shown in Figure 6, before adjusting for racial differences, the z-score distributions of normal subjects were separated between Koreans and Caucasians and then overlapped after the adjustment. The distribution of patients with AD also shows a similar pattern, to a lesser extent than in normal people. An important point to look at is the distance change between the distributions of Korean patients with AD and Caucasian normal controls. Their distributions were overlapped before the adjustment and then separated after the adjustment. Their differences in z-value before ethnicity adjustment were close to zero and then became clear after ethnicity adjustment (see Table 6, particularly the columns named “Kor.AD vs. Cau.CN”).

The result indicates that diagnostic errors could occur when doctors only diagnose Asian patients with AD with information from the Caucasian norms. They are likely to diagnose the patient as normal due to the highly overlapping distribution for Caucasian normal controls and Asian patients with AD before ethnicity adjustment.

**Figure 7:**
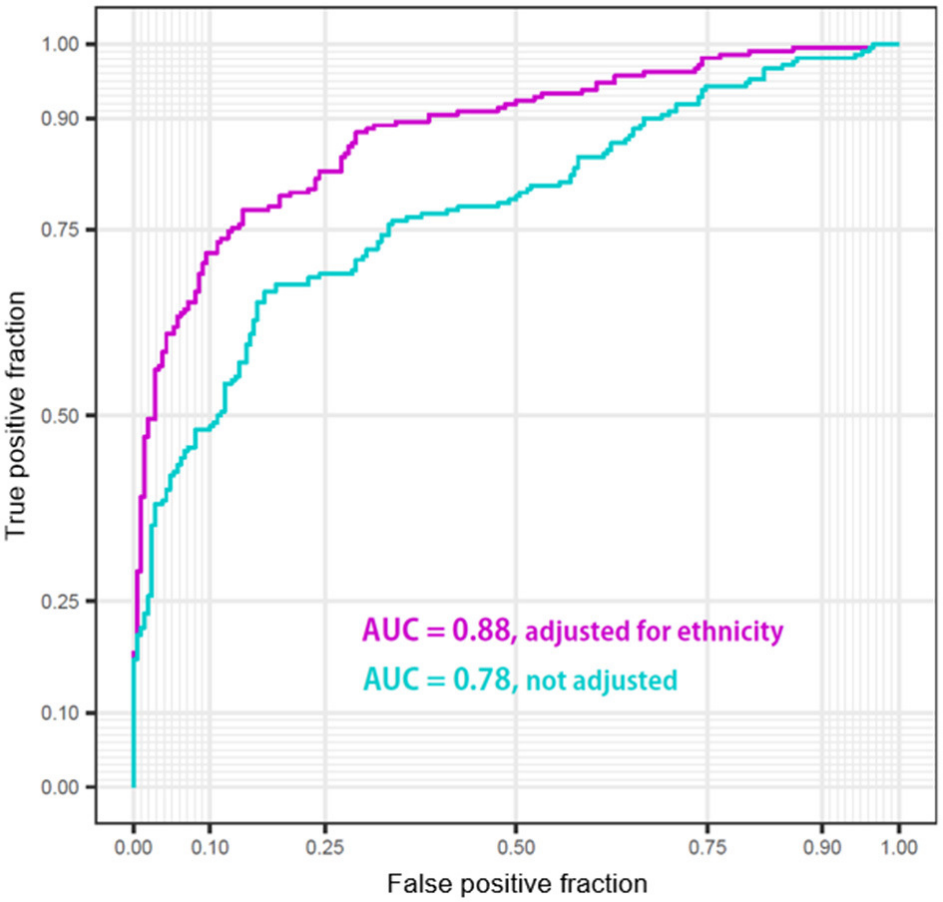
Performance of the classifiers of Korean patients Alzheimer's disease from Caucasian normal people using z-scores of bilateral temporal cortices, hippocampi, and amygdalae before/after adjustment for ethnic differences.

Finally, we analyzed whether and to what extent the ethnicity adjustment improved the diagnostic power of the logistic regression models built using the only z-scores of six regions: bilateral temporal cortices, hippocampi, and amygdalae. The performances of the classifier models were visualized as the two ROCs (Figure 7). The performance of the classifier after ethnicity adjustment (AUC = 0.88) was significantly improved compared with the classifier before ethnicity adjustment (AUC =0.78) (ΔAUC = 0.10, *D* = 7.80, *p* < 0.0001; AUC comparison test using bootstrap).

## Discussion

### General Summary

To our knowledge, the present study is the first to produce multi-racial normative volumes for lobar and subcortical structures in CN elderly individuals, considering ethnicity and age, sex, ICV, and characteristics of the MRI scanner using large samples restricted to old age. Even with the same number of samples, the sample dense in an age group can help the prediction model provide more reliable and precise estimates. The predictive model could be kept simple and non-over-fitted since the relationship between age and volume can be assumed to be linear, although hippocampal volumes were reported to be systemically overestimated to a less extent compared with young subjects when FreeSurfer measured (Wenger et al., 2014). The over-measurement is a function of ICV in elderly subjects. Since we included primary, secondary, and tertiary terms of ICV as predictors in our model, the z-scoring system can reduce the systematical errors caused by the over-measurement. Even if the inclusion of the terms of ICV could not work, the relative relationship between the two races will not change. Additionally, we focused on old age rather than whole life since comparing old and old will be more error-free than comparison between young and old.

We found that the temporal cortex, hippocampus, and amygdala were important for the AD diagnosis and highly influenced by the ethnic factor. Previous studies have shown that the temporal gyrus among cortical structures could be affected by some ethnic or genetic factors resulted in morphological differences such as brain shape or size (Zilles et al., 2001; Chee et al., 2011; Tang et al., 2018), possibly because the Asian brain is relatively wider than the Caucasian brain (Liang et al., 2015) and the temporal cortex is located on both sides of the brain. Our findings align with previous research reporting the significant effects of race or ethnicity on the hippocampus and amygdala volumes even in black and white children (Assari, 2020a,b). In contrast, ventricles and left pallidus did not significantly differ from the ethnicity in our result. Some studies reported the ethnicity effects on the ventricular and pallidal volumes in diverse racial comparisons such as Hispanic, African vs. white Americans (Minagar et al., 2000; Brickman et al., 2008), and Indigenous Australian vs. Caucasian women except men (Klekamp et al., 1989). The discrepancy with our results might be due to the different racial composition of the subjects in each study and the different aging slopes of the brain structures in each ethnic group that changed the order of the volumes dynamically across races according to age, i.e., Caucasians at relatively early ages showed larger volume than Asians but showed smaller volume at relatively late ages as shown in Figure 5 (Choi et al., 2020).

### Review/Comparison of Previous Studies

Ethnicity, as well as age, was found to affect brain volume significantly. All cortical structures were significantly greater in Koreans compared with Caucasians. Whole-brain size and ICV often has been used interchangeably. Even in old age, the whole brain volume is highly correlated to intracranial capacity (Pearson's *r* = 0.75, *p* < 0.001 for our whole sample). The larger the brain structures, the more proportional it tended to be to intracranial capacity. This tendency is reflected in the results that East Asian's largeness was noticeable, particularly in cortical or lobar volumes rather than in subcortical structures (Figures 3–5). Given that Caucasians had a little greater ICV (cf. Supplementary Table 1), the East Asian's largeness was rather surprising even after adjusted for ICV. One of the most probable explanations is that Koreans may have a high proportion of GM in the brain, judging from the finding that women show greater cortical GM thickness than men when adjusted for whole-brain size (Luders et al., 2006). Also, it might be due to brain morphometric differences between the ethnic groups, but the exact cause is unknown, and further research is needed.

There are, of course, research papers that take different stances from our data but not converging into one conclusion. Japanese hemispheres were reported to be wider but shorter than European hemispheres (Zilles et al., 2001). Compared with Caucasians, young Chinese men were observed to show larger volumes in temporal and cingulate cortices except for frontal and parietal lobes (Tang et al., 2018). Chinese Singaporeans and non-Asian Americans were not observed to be different for old groups, whereas the young Chinese group was found to have a lower cortical thickness in frontal, parietal, temporal lobes (Chee et al., 2011). All the previous studies involved just fewer than 70 persons as East Asians who are mostly young. The studies are inconsistent with each other. The superior size of our samples may have made the small ethnic difference more reliable.

### Limitations

Multi-study MRI analyses combining single- and multi-site datasets have limitations due to different scanner hardware and software versions and MRI protocols between studies. Even a multi-site study like ADNI uses dozens of protocols that contain many types of parameters such as TR, TE, flip angle, voxel size, and FoV. Furthermore, the process by which the complex interplay between the multiple factors affects images has not been clarified. The present study controlled two scanner-related variables: manufacturer and MFS, but not other scan parameters. We expected that the uncontrolled factors would play somewhat more the role of noise that increases variance than of bias that increases ethnic differences since the protocols and scanners used in ADNI were too diverse to produce a bias in a specific direction. As shown in Supplementary Figures 1, 2, the CIs, or variances, of the regression lines for ADNI were wider than those for GARD and OASIS that used only Siemens scanner, and the distances between the lines for ADNI and GARD were narrower than those for OASIS and GARD in most regions. These results made it less plausible that the ethnic differences we found were only due to different MRI protocols. According to Potvin et al. (2016), a combination of data from diverse sources is likely to provide more robust normative values than values generated using data from a single source, although there is a possibility to increase the noise or variance. Nevertheless, it is still necessary to collect brain images from manufacturers other than Siemens to enhance the diversity in the Korean sample.

All studies on humans have limitations in sampling, and our study is no exception. The Korean sample was based upon a population-based cohort in a city, whereas the Caucasian sample was based upon convenience cohorts collected by academic research groups. The GARD dataset can represent the Korean elderly because it is from a city where the prevalence of dementia and the per capita income are moderate in Korea. However, the ADNI and OASIS datasets can hardly represent the Caucasian elderly because of their cohort character. Although some datasets may not be strongly argued to represent the elderly population of their ethnic group, they are one of the largest samples in such studies comparing ethnic groups. Moreover, a study comparing a convenience sample and a population-based sample (Whitwell et al., 2012) reported that the differences in hippocampal volume between the two samples disappeared after matching for demographic information. Indeed, as shown in Figure 6, our validation procedure using the matched samples of healthy individuals showed that the z-score distributions overlapped between the two ethnic groups.

Although our study provides an insight into the normal aging of the brain, it has limitations due to its cross-sectional character. The limitations of cross-sectional and longitudinal studies were already discussed in our previous paper (Choi et al., 2020). However, the longitudinal studies have consistently supported the findings of the cross-sectional studies (Fjell et al., 2009), and brain atrophy is found to be greater in the longitudinal data than in cross-sectional data (e.g., Raz et al., 2005; Du et al., 2006; Taki et al., 2011; Fjell et al., 2014). Thus, the effects identified in the cross-sectional study would become more apparent in the longitudinal study, and a large sample-based cross-sectional study could explain a general trend of normal aging at a population level (Schuster et al., 2015).

Ethnicity or race is a very complex construct in which genetic and environmental factors are mixed. So, we do not argue that the observed differences in brain volumes were only caused by genetic background. Alternative explanations involving environmental as well as genetic sub-factors of ethnicity should be considered. For example, since obesity, a cardiovascular risk factor that may cause brain structure atrophy (Hamer and Batty, 2019; Opel et al., 2020), ethnic differences in obesity measures like body mass index could explain the ethnic differences in brain volumes. The obesity of an ethnic group is related to their dietary culture, which can be considered as an environmental component of ethnicity. However, in the present study, such sub-factors of ethnicity were not rigorously controlled because they could be broadly viewed as constituent elements in the concept of ethnicity. Strictly speaking, our study is not about identifying brain regions affected only by genetic components of ethnicity but rather revealing ethnic norms in brain volume and inventing methods to reduce the discrepancies between the norms at the current time. Further research is needed to dissect which factors cause the ethnic differences in norms. In future studies, the interplay between genetic and environmental factors in ethnicity that affects aging deserves more attention.

## Conclusion

This normative data for the aging brain considering ethnic backgrounds can render researchers and clinicians with the age-related reference ranges needed to facilitate research and precise diagnosis of degenerative brain diseases in diverse ethnic societies.

## Data Availability Statement

The datasets presented in this study can be found in online repositories. The names of the repository/repositories and accession number(s) can be found below: ADNI dataset (https://adni.loni.usc.edu) and the OASIS-3 dataset (https://www.oasis-brains.org/). The GARD data that support the findings of this study are not openly available yet. Until we are ready to share the data publicly, the data could be available from the corresponding author (BCK or KHL), upon reasonable request.

## Ethics Statement

The studies involving human participants were reviewed and approved by the Institutional Review Board of Chosun University Hospital, Gwangju, Republic of Korea. The patients/participants provided their written informed consent to participate in this study.

## Author Contributions

YYC performed the imaging analysis. YYC and U-SC wrote the preliminary draft. YYC, JJL, and KYC conducted the statistical analysis. EHS, IHC, M-KS, and YC interpreted the neuropsychological results. HK, S-MC, SHC, and BCK interviewed and examined the patients/participants and read the brain MRIs. BCK and KHL designed the study, wrote the manuscript, and provided the overall supervision for the project. All authors contributed to the article and approved the submitted version.

## Conflict of Interest

KHL was employed by the company Neurozen Inc. The remaining authors declare that the research was conducted in the absence of any commercial or financial relationships that could be construed as a potential conflict of interest.

## Publisher's Note

All claims expressed in this article are solely those of the authors and do not necessarily represent those of their affiliated organizations, or those of the publisher, the editors and the reviewers. Any product that may be evaluated in this article, or claim that may be made by its manufacturer, is not guaranteed or endorsed by the publisher.

## Supplementary Material

Supplementary Figure 1 Lobar volume changes in Caucasian (ADNI and OASIS datasets) and Mongolian elderly peoples (GARD dataset). This figure illustrates ethnic contrast in age effect in each model predicting lobar volumes in a massive sample of cognitively normal people aged 59–89 years. Each line denotes mean volume with 95% confidence intervals in the colored shade.

Supplementary Figure 2 Subcortical volume changes in Caucasian (ADNI and OASIS datasets) and Mongolian elderly peoples (GARD dataset). This figure illustrates ethnic contrast in age effect in each model predicting subcortical volumes in a massive sample of cognitively normal people aged 59–89 years. Each line denotes mean volume with 95% confidence intervals in the colored shade. Ventricular volumes are log_10_ transformed.

Supplementary Table 1 Intracranial volume (ICV) for normal Koreans and Caucasians.

Supplementary Table 2 Standardized coefficients of the prediction model including the three-way interaction term: MFS x ICV x manufacturer.

Supplementary Table 3 Standardized coefficients of the prediction model using the image data of Siemens 3T scanners only.
