# Supplementary material for: Multi-Racial Normative Data for Lobar and Subcortical Brain Volumes in Old Age: Korean and Caucasian Norms May Be Incompatible With Each Other†
Source: Front Aging Neurosci. 2021 Aug 3;13:675016. doi: 10.3389/fnagi.2021.675016 (PMC8369368; doi:10.3389/fnagi.2021.675016)
Supplement: Supplementary Figure 1 — Lobar volume changes in Caucasian (ADNI and OASIS datasets) and Mongolian elderly peoples (GARD dataset). This figure illustrates ethnic contrast in age effect in each model predicting lobar volumes in a massive sample of cognitively normal people aged 59–89 years. Each line denotes mean volume with 95% confidence intervals in the colored shade. [file Image_1.pdf]

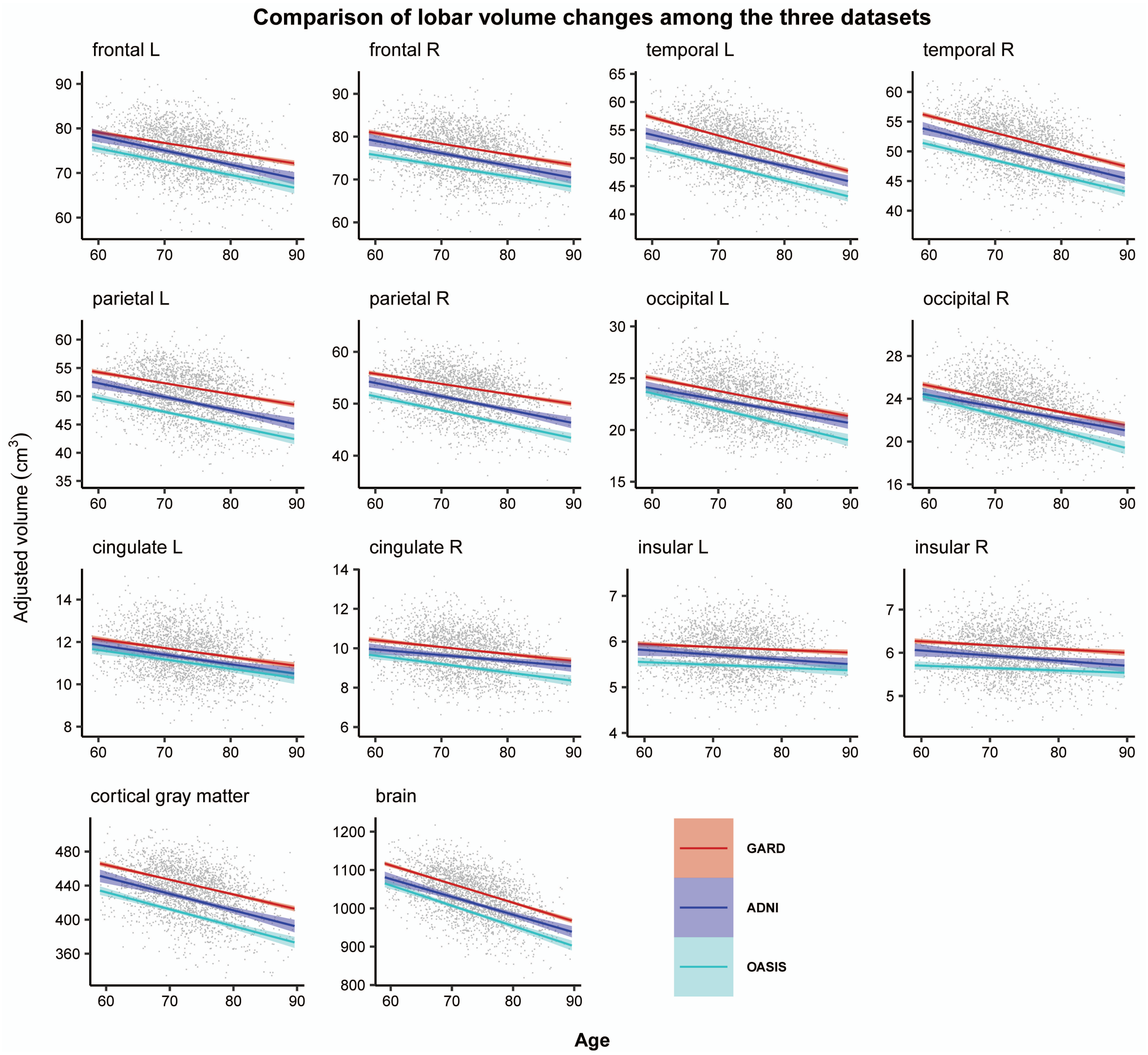

Supplementary Figure 1. Lobar volume changes in Caucasian (ADNI and OASIS datasets) and Mongolian elderly peoples (GARD dataset). This figure illustrates ethnic contrast in age effect in each model predicting lobar volumes in a massive sample of cognitively normal people aged 59–89 years. Each line denotes mean volume with 95% confidence intervals in colored shade.
